# AaSnRK2.6 interacts with AaORA to positively regulate artemisinin biosynthesis in *Artemisia annua*

**DOI:** 10.1093/hr/uhag287

**Published:** 2026-07-07

**Authors:** Guoping Shu, Fei Su, Yueli Tang, Junnan Li, Mohammad Mahmoud Nagdy, Lingjiang Zeng, Fangyuan Zhang, Min Chen, Jingxin Mao, Zhihua Liao

**Affiliations:** Guizhou Key Laboratory for Germplasm Innovation and Resource-Efficient Utilization of Dao-di Herbs, Guizhou University of Traditional Chinese Medicine, Guiyang 550025, China; Integrative Science Center of Germplasm Creation in Western China (CHONGQING) Science City, Southwest Bio-resources R&D Key Laboratory of Sichuan Province, SWU-TAAHC Medicinal Plant Joint R&D Centre, School of Life Sciences, Southwest University, Chongqing 400715, China; Integrative Science Center of Germplasm Creation in Western China (CHONGQING) Science City, Southwest Bio-resources R&D Key Laboratory of Sichuan Province, SWU-TAAHC Medicinal Plant Joint R&D Centre, School of Life Sciences, Southwest University, Chongqing 400715, China; Integrative Science Center of Germplasm Creation in Western China (CHONGQING) Science City, Southwest Bio-resources R&D Key Laboratory of Sichuan Province, SWU-TAAHC Medicinal Plant Joint R&D Centre, School of Life Sciences, Southwest University, Chongqing 400715, China; Integrative Science Center of Germplasm Creation in Western China (CHONGQING) Science City, Southwest Bio-resources R&D Key Laboratory of Sichuan Province, SWU-TAAHC Medicinal Plant Joint R&D Centre, School of Life Sciences, Southwest University, Chongqing 400715, China; College of Pharmaceutical Sciences, Southwest University, Chongqing 400715, China; Department of Medicinal and Aromatic Plants Research, National Research Centre, Dokki, Cairo 12311, Egypt; Integrative Science Center of Germplasm Creation in Western China (CHONGQING) Science City, Southwest Bio-resources R&D Key Laboratory of Sichuan Province, SWU-TAAHC Medicinal Plant Joint R&D Centre, School of Life Sciences, Southwest University, Chongqing 400715, China; Integrative Science Center of Germplasm Creation in Western China (CHONGQING) Science City, Southwest Bio-resources R&D Key Laboratory of Sichuan Province, SWU-TAAHC Medicinal Plant Joint R&D Centre, School of Life Sciences, Southwest University, Chongqing 400715, China; College of Pharmaceutical Sciences, Southwest University, Chongqing 400715, China; Chongqing Municipal Key Laboratory for High Active Traditional Chinese Drug Delivery System, Chongqing Medical and Pharmaceutical College, Chongqing 401331, China; Integrative Science Center of Germplasm Creation in Western China (CHONGQING) Science City, Southwest Bio-resources R&D Key Laboratory of Sichuan Province, SWU-TAAHC Medicinal Plant Joint R&D Centre, School of Life Sciences, Southwest University, Chongqing 400715, China

## Abstract

Artemisinin, a sesquiterpene lactone produced in *Artemisia annua*, is the most effective antimalarial compound, but its low natural abundance in *A. annua* remains a major bottleneck for large-scale production. Increasing its biosynthesis requires a better understanding of the regulatory networks controlling artemisinin biosynthesis genes. Several jasmonate (JA)-responsive transcription factors have been characterized, but the role of upstream kinases remains poorly understood. Here, we identify AaSnRK2.6, a SnRK2III-type protein kinase, as a novel positive regulator of artemisinin biosynthesis. *AaSnRK2.6* expression was strongly induced by methyl jasmonate and abscisic acid, and the protein is localized to both the cytoplasm and nucleus. Protein interaction assays revealed that AaSnRK2.6 specifically interacts with the JA-responsive TF AaORA in the nucleus, and an *in vitro* kinase assay further showed that AaSnRK2.6 directly phosphorylates AaORA. Dual-luciferase assays showed that AaSnRK2.6 enhances AaORA-mediated activation of key artemisinin biosynthetic genes (amorpha-4,11-diene synthase, *DBR2*, and *ALDH1*). Overexpression of *AaSnRK2.6* alone or *AaORA* alone significantly increased artemisinin and dihydroartemisinic acid accumulation, while co-overexpression of *AaSnRK2.6* and *AaORA* in transgenic plants produced the stronger effect. By contrast, RNA interference knockdown of *AaSnRK2.6* showed no significant effect on artemisinin levels, likely due to functional redundancy of kinases. Collectively, this study supports a positive kinase-TF regulatory module involved in hormone-responsive artemisinin biosynthesis in *A. annua* and provides new insights into its regulatory mechanism.

## Introduction

Artemisinin is a sesquiterpene lactone characterized by a unique endoperoxide bridge. Recognized as the most effective anti-malarial compound identified to date, artemisinin has been credited with saving millions of lives worldwide, particularly in malaria-endemic regions [1, 2]. Owing to its potent activity against *Plasmodium falciparum*, artemisinin and its semi-synthetic derivatives have been officially recommended by the World Health Organization as first-line antimalarial drugs [3, 4]. Although artemisinin can be chemically synthesized from artemisinic acid, such approaches are often labor-intensive and costly, making plant-derived extraction the primary commercial source. Unfortunately, *Artemisia annua* plants accumulate relatively low levels of artemisinin, typically ranging from 0.1% to 1% of leaf dry weight [5], which poses a significant limitation on natural extraction yields.

To date, the complete biosynthetic pathway of artemisinin has been elucidated. Like most terpenoids, artemisinin is derived from farnesyl pyrophosphate (FPP), which itself is synthesized through the condensation of isopentenyl pyrophosphate (IPP) and dimethylallyl pyrophosphate, a reaction catalyzed by farnesyl pyrophosphate synthase [6, 7]. FPP undergoes a series of enzymatic transformations to produce dihydroartemisinic acid (DHAA) and ultimately artemisinin (AA) [8]. Key enzymes involved in this process include amorpha-4,11-diene synthase (ADS), cytochrome P450 monooxygenase (CYP71AV1), artemisinic aldehyde Δ11 reductase (DBR2), and aldehyde dehydrogenase 1 (ALDH1) [9–13]. These enzymes represent key rate-limiting steps and are predominantly expressed in the glandular secretory trichomes (GSTs) of *A. annua*, critically determining biosynthetic efficiency [14]. The artemisinin biosynthetic pathway in *A. annua* plants is intricately regulated by multiple plant hormones and environmental cues [15]. Among them, jasmonic acid (JA) and abscisic acid (ABA) are prominent positive regulators. Exogenous applications of methyl jasmonate (MeJA) or ABA significantly elevate the expression of key enzyme genes such as *ADS*, *CYP71AV1*, *DBR2*, and *ALDH1*, thereby enhancing artemisinin accumulation in *A. annua* plants [16, 17]. While MeJA activates a suite of jasmonate-responsive transcription factors (TFs) that form complex regulatory networks controlling artemisinin biosynthesis [18, 19], the detailed molecular interplay between JA and ABA signaling in this context remains insufficiently understood.

The sucrose nonfermenting 1-related protein kinases (SnRKs) constitute a conserved family of serine/threonine protein kinases in plants, classified into SnRK1, SnRK2, and SnRK3 subfamilies [20]. Among them, SnRK2s are unique to plants and act as central positive regulators of ABA signaling by phosphorylating downstream TFs to activate stress-responsive gene expression and metabolic adaptation [21]. Emerging evidence indicates that SnRK2s also participate in cross-communication between ABA and other hormonal pathways. In rice, ABA-activated SnRK2s phosphorylate TFs involved in JA biosynthesis, integrating defense and developmental programs [22, 23]. Mechanistically, SnRK2-mediated phosphorylation often modulates TF stability, subcellular localization, or DNA-binding affinity, providing a rapid layer of regulation beyond transcriptional control [24]. However, it remains unclear whether SnRK2s participate in JA-induced artemisinin biosynthesis.

Increasing evidence highlights the significance of post-translational modifications, particularly phosphorylation of TFs, in fine-tuning plant secondary metabolism. Examples include tyrosine kinase-mediated phosphorylation of WRKY to regulate berberine biosynthesis in *Coptis japonica* [25], MAPK-dependent phosphorylation of ORCAs controlling terpenoid indole alkaloid production in *Catharanthus roseus* [26], and MPK4-mediated stabilization of MYB75 driving anthocyanin biosynthesis in *Arabidopsis* [27]. In medicinal plants, *Salvia miltiorrhiza* SnRK2.6 enhances phenolic acid accumulation by phosphorylating SmAREB1 [28], while in *A. annua*, AaAPK1 activates AabZIP1 to boost artemisinin levels [29]. These findings underscore the critical role of kinase-mediated modulation of TFs beyond transcriptional control alone. AaSnRK2.6 identified in this study is distinct from the previously reported AaAPK1 and was named according to its closest phylogenetic relationship with AtSnRK2.6.

In *A. annua*, the AP2/ERF TF AaORA is a pivotal regulator that integrates JA and ABA signals to upregulate key artemisinin biosynthetic genes [30, 31]. AaORA synergistically interacts with AaTCP14 and AaTCP15 to activate promoters of key genes such as *DBR2* and *ALDH1*, while also enhancing AaTCP15 activity and expression [30, 32]. Although this JA-ABA convergence via TF cooperation has been established, it remains unknown whether upstream kinases participate by phosphorylating AaORA or its partners, thereby fine-tuning the regulatory output.

In this study, we identified AaSnRK2.6, a SnRK2III kinase in *A. annua*, as a positive regulator of artemisinin biosynthesis. We demonstrate that AaSnRK2.6 expression is induced by MeJA and that it physically interacts with AaORA in the nucleus. Functional analyses revealed that AaSnRK2.6 enhances AaORA-mediated activation of key biosynthetic genes, and co-overexpression of *AaSnRK2.6* and *AaORA* significantly increased artemisinin and dihydroartemisinic acid accumulation. Our findings reveal a novel kinase-TF regulatory module that links JA signaling to artemisinin biosynthesis, offering new opportunities for metabolic engineering in medicinal plants.

## Results

### Characterization of AaSnRK2.6 in *A. annua*

**Figure 1:**
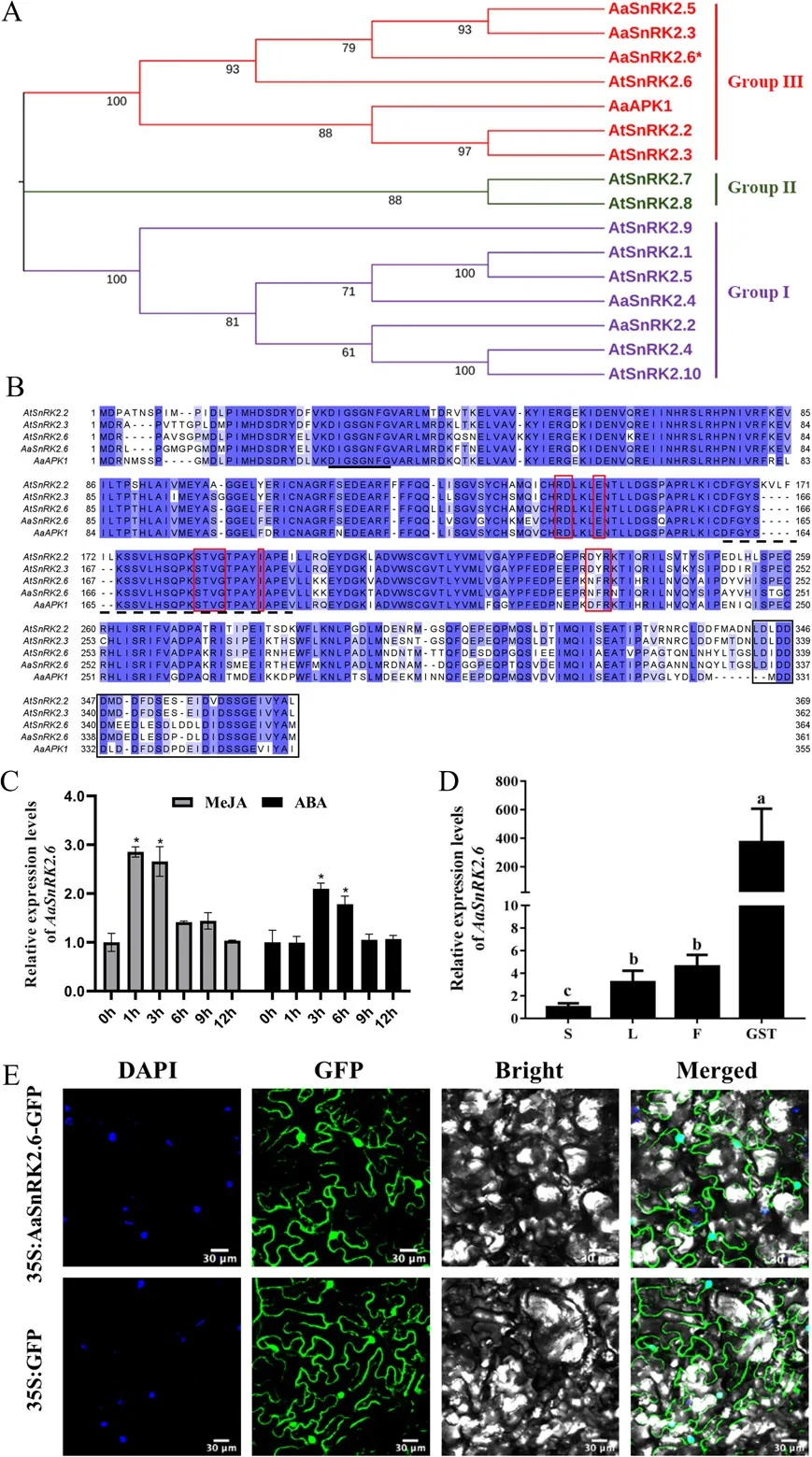
Phylogenetic characterization, conserved domain analysis, expression patterns, and subcellular localization of AaSnRK2.6 in *Artemisia annua*. (A) Phylogenetic analysis of AaSnRK2.6 together with 10 SnRK2 protein kinases from *Arabidopsis thaliana*. The lower, middle, and upper clades correspond to SnRK2 subfamilies I, II, and III, respectively, as indicated by the labels on the right. Bootstrap values (%) are indicated at the corresponding nodes of the maximum-likelihood tree. (B) Multiple sequence alignment of AaSnRK2.6, AtSnRK2.2, AtSnRK2.3, AtSnRK2.6, and AaAPK1. Identical residues are highlighted in black on a blue background. The black solid line indicates the ATP-binding loop, the black dashed line denotes the activation loop, and the red box marks the PP2C-interacting residues. (C) Relative expression of AaSnRK2.6 in response to 10 μM ABA and 100 μM MeJA treatments. *β*-actin served as the internal reference gene. Data are presented as mean ± SD (*n* = 3). *P* < 0.05; *P* < 0.01 (Student’s *t*-test). (D) Tissue-specific expression of *AaSnRK2.6* in *A. annua*. S, stem; L, leaf; F, flower; GST, glandular secretory trichomes. Different letters indicate significant differences (Duncan’s multiple range test, *n* = 3). (E–F) Subcellular localization of AaSnRK2.6 in *N. benthamiana* epidermal cells. (E) 35S:*AaSnRK2.6*-GFP; (F) 35S:GFP control. DAPI, nuclear stain; GFP, green fluorescent protein signal; Bright, bright-field image; Merged, overlay of DAPI, GFP, and bright-field images. Scale bar = 30 μm.

A genome-wide search of the *A. annua* transcriptome identified five putative SnRK2 genes based on HMM profiles and sequence similarity to the *Arabidopsis thaliana* SnRK2 family. Among them, one full-length gene was successfully cloned, containing a 1086 bp coding sequence that encodes a 362-amino-acid polypeptide (Supplementary Fig. S1). Phylogenetic analysis showed that this *A. annua* gene clustered within subfamily III and was most closely related to AtSnRK2.6 (Fig. 1A). It was therefore designated AaSnRK2.6. This gene is distinct from the previously reported AaAPK1, which represents another SnRK2 family member in *A. annua*. It was therefore designated AaSnRK2.6. Multiple sequence alignment further revealed that AaSnRK2.6 shares 87.3% amino acid identity with AtSnRK2.6 and retains the conserved ATP-binding site, kinase catalytic domain, ABA box, and key residues required for PP2C interaction, indicating that AaSnRK2.6 is a typical member of the SnRK2 family (Fig. 1B).

qRT-PCR analysis showed that *AaSnRK2.6* transcript levels were markedly induced by MeJA and also responded to ABA, with both treatments causing rapid upregulation at early time points (Fig. 1C). Tissue-specific expression analysis further showed that *AaSnRK2.6* was expressed in multiple tissues but accumulated most abundantly in GSTs (Fig. 1D), which are the primary site of artemisinin biosynthesis in *A. annua*. This expression pattern suggests that AaSnRK2.6 may participate in the regulation of artemisinin biosynthesis.

Transient expression of 35S:AaSnRK2.6-GFP in *N. benthamiana* epidermal cells showed that the fusion protein was localized to both the nucleus and cytoplasm, whereas free GFP was distributed throughout the cell (Fig. 1E–F). This dual localization suggests that AaSnRK2.6 may function in both cytoplasmic signal transduction and nuclear regulatory processes. In addition, MeJA-induced expression of *ADS*, *DBR2*, and *ALDH1*, as well as artemisinin accumulation, was enhanced in *AaSnRK2.6*-overexpression lines, providing further support for a positive role of AaSnRK2.6 in MeJA-responsive artemisinin biosynthesis (Supplementary Fig. S2).

### AaSnRK2.6 specifically interacts with AaORA

**Figure 2:**
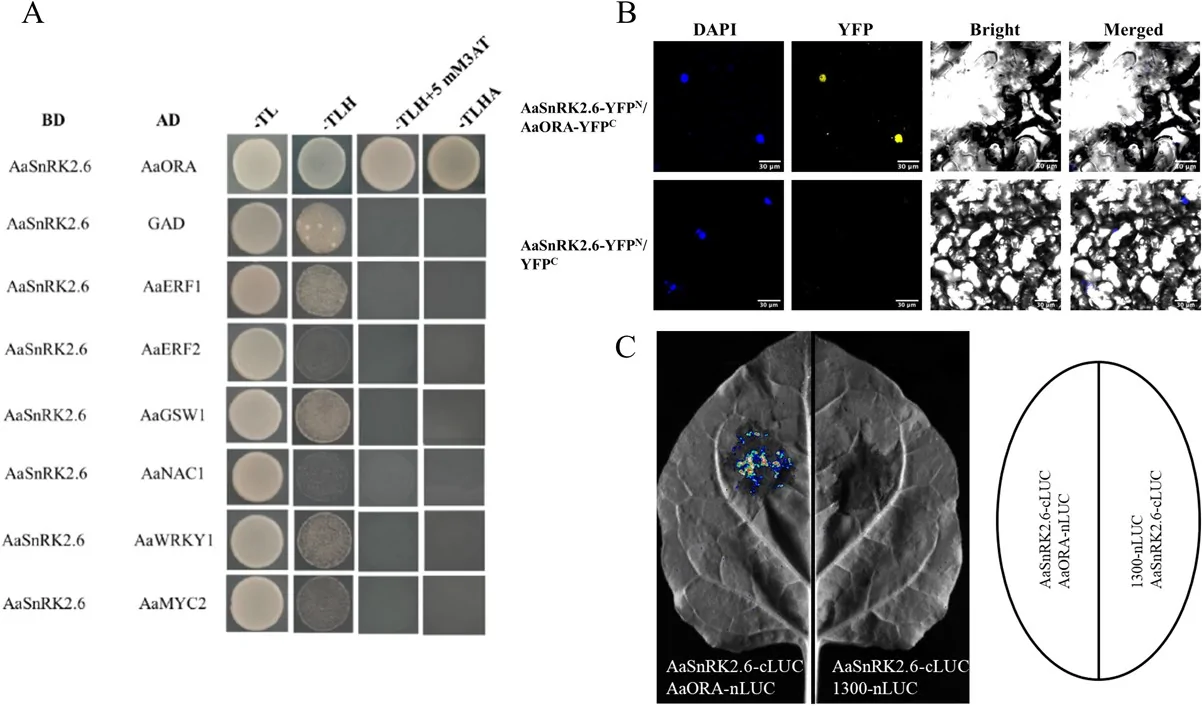
AaSnRK2.6 specifically interacts with AaORA. (A) Yeast two-hybrid (Y2H) assay showing the specific interaction between AaSnRK2.6 and AaORA. BD: DNA-binding domain; AD: activation domain; -TL: SD/-Trp/-Leu; -TLH: SD/-Trp/-Leu/-His; -TLHA: SD/-Trp/-Leu/-His/-Ade. (B) BiFC assay confirming the interaction in tobacco cells. DAPI: nuclear stain; YFP: yellow fluorescent protein signal. (C) LCI assay validating the interaction in tobacco cells.

SnRK2 kinases in plants typically undergo reversible phosphorylation within their activation loops and subsequently phosphorylate downstream TFs to mediate stress or hormone signaling. Considering that *AaSnRK2.6* expression was strongly induced by MeJA, we hypothesized that it might participate in JA signaling by interacting with key TFs involved in artemisinin biosynthesis. To test this, we cloned several JA-responsive TFs implicated in regulating artemisinin biosynthesis, including AaGSW1, AaWRKY1, AaORA, AaERF1, AaERF2, AaNAC1, AabHLH112, and AaMYC2 [15], into the pGADT7 vector, while AaSnRK2.6 was cloned into pGBKT7. Yeast two-hybrid (Y2H) assays showed that, although pGBKT7-*AaSnRK2.6* exhibited slight self-activation on SD/-Trp/-Leu/-His (-TLH) medium, this was effectively suppressed by the addition of 5 mM 3-AT. Under these stringent conditions, only yeast co-expressing *AaSnRK2.6* and *AaORA* grew robustly on SD/-TLH + 5 mM 3-AT and SD/-Trp/-Leu/-His/-Ade (-TLHA) media, whereas no growth was observed for other combinations (Fig. 2A), indicating a specific interaction between AaSnRK2.6 and AaORA.

To further validate this interaction in *planta*, Bimolecular fluorescence complementation (BiFC) assays were performed in *N. benthamiana* leaves. Co-expression of *AaORA* fused to the N-terminal fragment of YFP (YFP^N^) and *AaSnRK2.6* fused to the C-terminal fragment (YFP^C^) generated strong YFP fluorescence localized exclusively in the nucleus, overlapping with DAPI staining. No fluorescence was observed in the negative controls (Fig. 2B). This result supported their nuclear interaction. Because BiFC can occasionally yield false positives due to nonspecific reconstitution, luciferase complementation imaging (LCI) assays were also conducted. Co-expression of *AaSnRK2.6*-cLUC and *AaORA*-nLUC in *N. benthamiana* leaves produced strong luminescence signals, whereas control combinations showed no detectable luminescence (Fig. 2C), conclusively confirming the specific interaction between AaSnRK2.6 and AaORA.

### AaSnRK2.6 enhances AaORA-mediated activation of artemisinin biosynthetic genes

**Figure 3:**
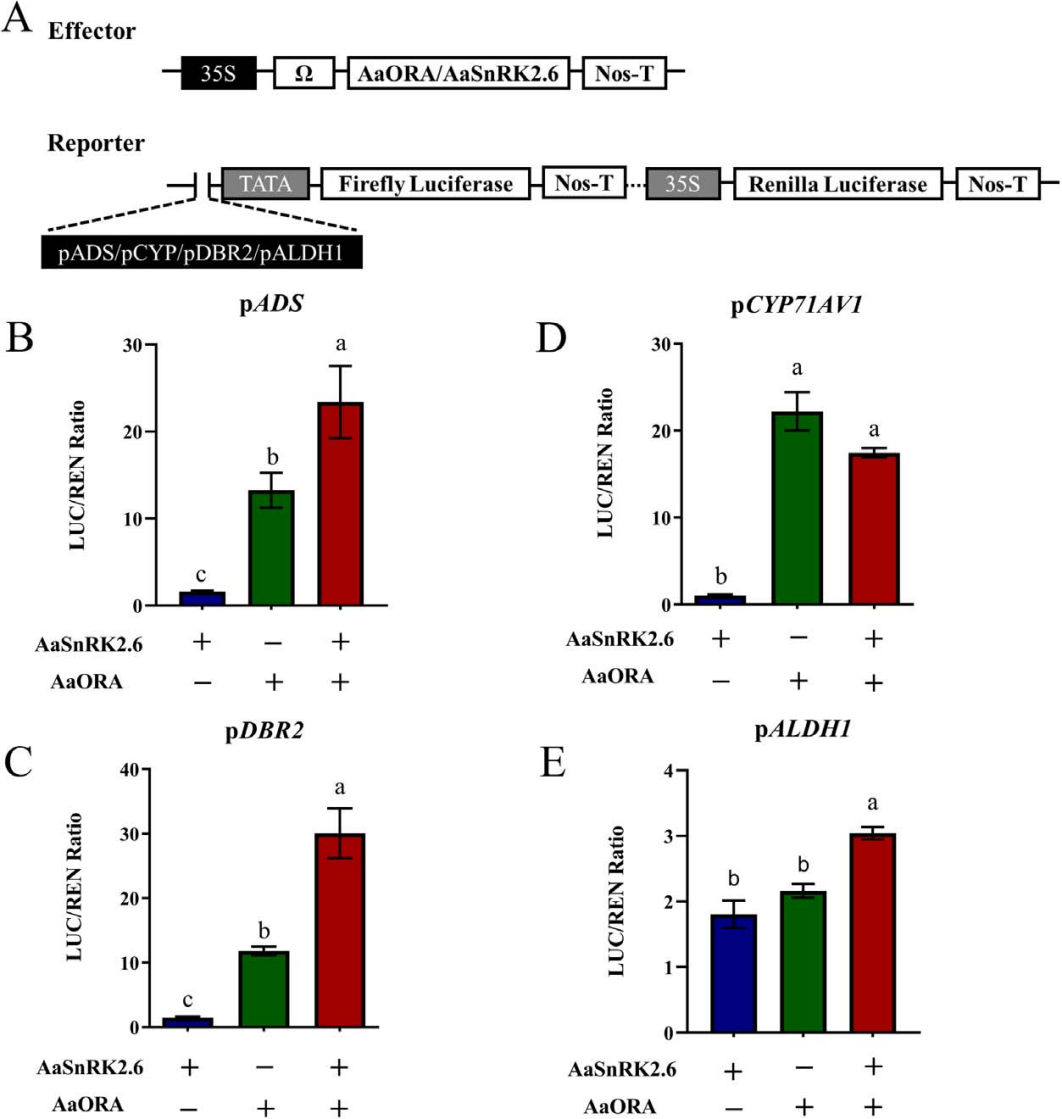
AaSnRK2.6 enhances AaORA-mediated transcriptional activation of artemisinin biosynthetic genes. (A) Diagram of effector and reporter constructs used in the dual-luciferase assay. (B-E) Effects of AaSnRK2.6, AaORA, and their co-expression on the promoter activities of key artemisinin biosynthetic genes (*ADS*, *CYP71AV1*, *DBR2*, and *ALDH1*). Luciferase activity (LUC/REN ratio) was normalized to that of the empty-vector control, which represents the baseline promoter activity. ‘+’ indicates presence of the corresponding effector protein; ‘–’ indicates absence. Different letters indicate significant differences (one-way ANOVA followed by Tukey’s HSD test, *n* = 3, *P* < 0.05).

To investigate whether the interaction between AaSnRK2.6 and AaORA affects the transcriptional regulation of key artemisinin biosynthetic genes, dual-luciferase assays were performed in *N. benthamiana*. Promoter fragments of four key artemisinin biosynthetic genes (*ADS*, *CYP71AV1*, *DBR2*, and *ALDH1*) were fused to the firefly luciferase reporter in the pGreen II-0800 vector, while *AaSnRK2.6* and *AaORA* were individually cloned into the CaMV35S-driven pHB effector vector (Fig. 3A). Co-infiltration with the empty pHB-GFP vector served as the negative control. The promoter activities shown in Fig. 3 are expressed as relative LUC/REN ratios normalized to the empty-vector control, which represents the baseline promoter activity.

Transient expression of *AaSnRK2.6* alone did not significantly affect the activity of any tested promoters, whereas *AaORA* alone strongly activated *proADS*, *proCYP71AV1*, and *proDBR2* by 13.27-, 21.22-, and 2.16-fold respectively, relative to the control. Remarkably, co-expression of *AaSnRK2.6* with *AaORA* further enhanced the activities of *proADS*, *proDBR2*, and *proALDH1* by 1.76-, 2.54-, and 1.41-fold respectively, compared with *AaORA* alone, whereas *proCYP71AV1* activity remained largely unchanged (Fig. 3B–E). These results indicate that AaSnRK2.6 specifically enhances the transcriptional activation capability of AaORA on a subset of key artemisinin biosynthetic genes, suggesting a synergistic role of the two proteins in the transcriptional regulation of artemisinin metabolism.

### AaSnRK2.6 interacts with and directly phosphorylates AaORA *in vitro*

**Figure 4:**
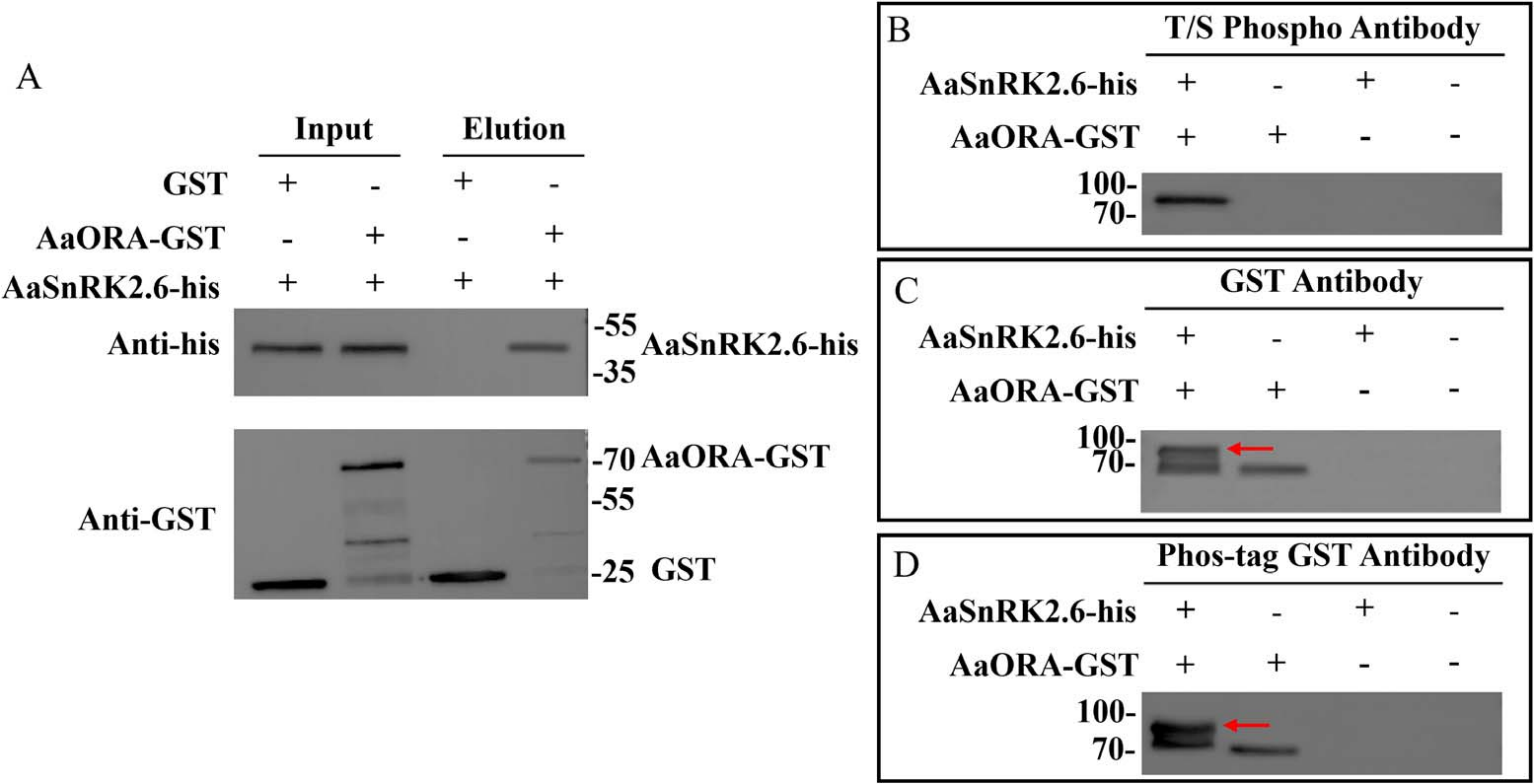
AaSnRK2.6 interacts with and directly phosphorylates AaORA *in vitro*. (A) GST pull-down assay showing the *in vitro* interaction between AaSnRK2.6-His and AaORA-GST. GST was used as a negative control. Input and elution fractions were detected by immunoblotting with anti-His and anti-GST antibodies. (B) *In vitro* kinase assay showing the phosphorylation of AaORA-GST by AaSnRK2.6-His, detected with an anti-phosphoserine/threonine antibody. (C) Immunoblot analysis with an anti-GST antibody confirming the presence of AaORA-GST in the corresponding reaction mixtures. (D) Phos-tag detection further supporting the phosphorylation of AaORA-GST by AaSnRK2.6-His. ‘+’ and ‘−’ indicate the presence or absence of the corresponding protein in each reaction.

To further examine whether AaSnRK2.6 directly acts on AaORA at the protein level, we first performed a GST pull-down assay using recombinant proteins. In the elution fractions, AaSnRK2.6-His was detected only in the presence of AaORA-GST, but not with GST alone, indicating a specific *in vitro* interaction between AaSnRK2.6 and AaORA (Fig. 4A). We next carried out an *in vitro* kinase assay using recombinant AaSnRK2.6-His and AaORA-GST proteins. Immunoblot analysis with an anti-phosphoserine/threonine antibody detected a clear phosphorylation signal only in the reaction containing both proteins, whereas the corresponding control reactions did not show such a signal (Fig. 4B). Immunoblotting with an anti-GST antibody confirmed the presence of AaORA-GST in the corresponding reaction mixtures (Fig. 4C). Furthermore, Phos-tag detection revealed a mobility shift consistent with phosphorylation of AaORA-GST in the presence of AaSnRK2.6-His (Fig. 4D). Together, these results demonstrate that AaSnRK2.6 can physically associate with and directly phosphorylate AaORA *in vitro*.

### Co-overexpression of *AaSnRK2.6* and *AaORA* increases artemisinin accumulation

**Figure 5:**
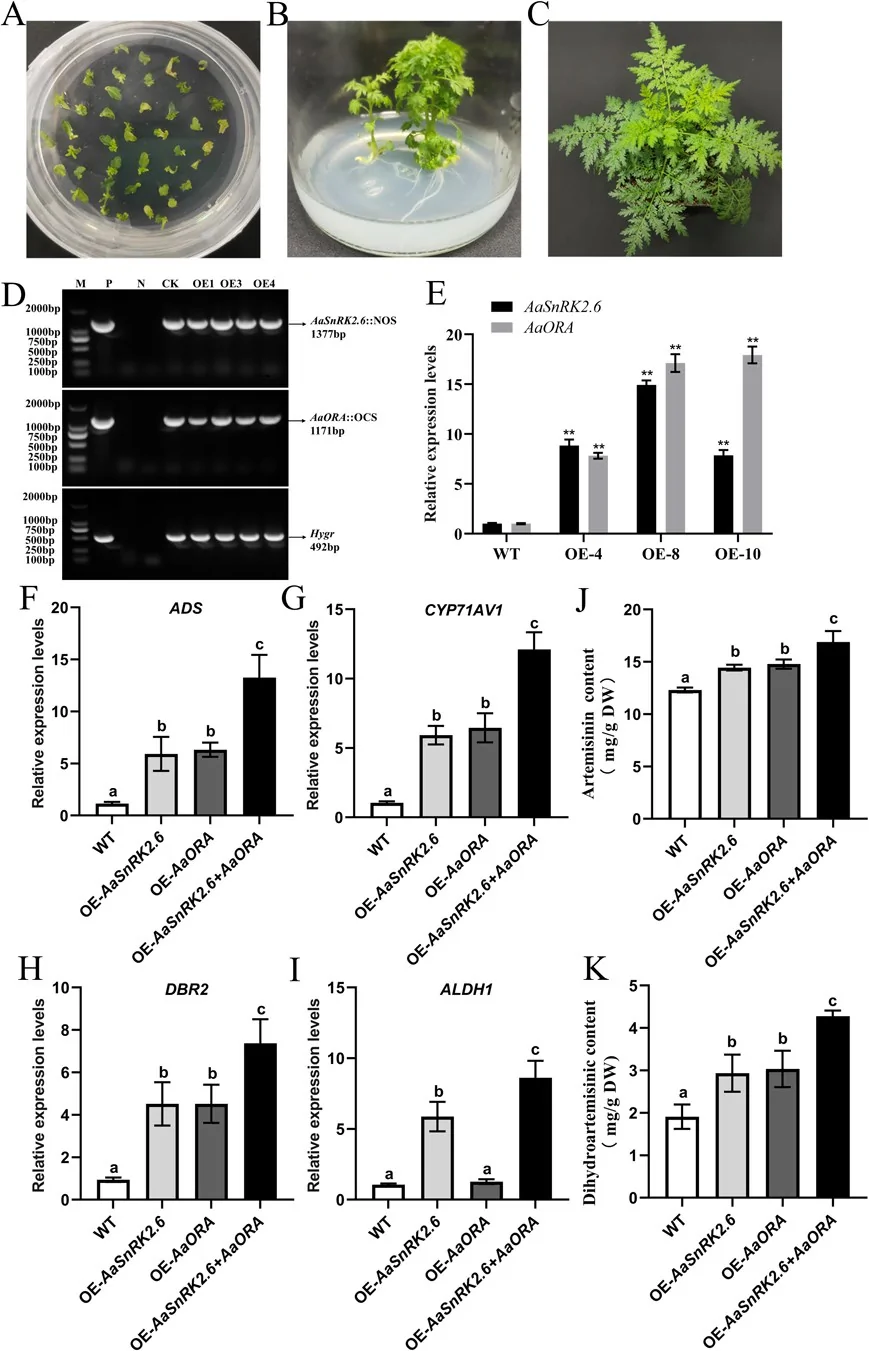
Overexpression of *AaSnRK2.6* alone and co-overexpression of *AaSnRK2.6* with *AaORA* promote artemisinin biosynthesis in transgenic *A. annua*. (A–C) Regeneration of transgenic *A. annua* overexpressing *AaSnRK2.6*: hygromycin resistance screening (A), induction of roots (B), and regenerated transgenic plant (C). (D) Genomic PCR verification of transgenic *A. annua* co-overexpressing *AaSnRK2.6* and *AaORA*. M: 2000 bp DNA marker; P: positive control; N: negative control; CK: blank control. (E) Relative expression levels of *AaSnRK2.6* and *AaORA* in transgenic lines. WT: wild-type; *β*-actin was used as an internal reference. Data are presented as means ± SD (*n* = 9). ^*^P < 0.05; ^**^P < 0.01 (Student’s *t*-test). (F–I) Relative expression levels of key artemisinin biosynthetic genes (*ADS*, *CYP71AV1*, *DBR2*, and *ALDH1*) in WT, *AaSnRK2.6*-overexpression (OE-*AaSnRK2.6*), *AaORA*-overexpression (OE-*AaORA*), and *AaSnRK2.6*/*AaORA* co-overexpression (OE-*AaSnRK2.6*-*AaORA*) lines. (J-K) Artemisinin and DHAA contents in WT, OE-*AaSnRK2.6*, OE-*AaORA*, and OE-*AaSnRK2.6*-*AaORA* lines. Data are presented as mean ± SD (*n* = 9). Different letters indicate significant differences according to one-way ANOVA followed by Tukey’s HSD test (*P* < 0.05).

To validate whether AaSnRK2.6 promotes artemisinin biosynthesis by potentiating the transcriptional activity of AaORA in *A. annua*, a dual-gene co-expression construct (pZZG100-*AaSnRK2.6*-*AaORA*) was generated and introduced into *A. annua* via *Agrobacterium*-mediated transformation. During regeneration, transformed leaf explants produced shoots on selective medium (Fig. 5A), developed roots on rooting medium (Fig. 5B), and were successfully acclimatized to soil (Fig. 5C). Genomic PCR using gene-specific and vector-derived primers (*NOS*-R, *OCS*-R) confirmed stable integration of both transgenes, with expected bands detected in transgenic lines but absent in WT plants (Fig. 5D).

Three representative dual overexpression lines (OE-4, OE-8, OE-10) exhibiting high transcript levels were selected for further analysis. qPCR confirmed significantly elevated expression of *AaSnRK2.6* and *AaORA* in these lines relative to WT (Fig. 5E). Consistently, the transcript levels of *ADS*, *CYP71AV1*, *DBR2*, and *ALDH1* were also markedly upregulated in the co-overexpression lines. Specifically, in *AaSnRK2.6* single overexpression lines, the expression levels of *ADS*, *CYP71AV1*, *DBR2*, and *ALDH1* were increased by 4.91-, 4.93-, 3.52-, and 5.77-fold respectively compared to the WT control, whereas *AaORA* single overexpression lines showed 5.29-, 5.46-, and 3.52-fold increases for *ADS*, *CYP71AV1*, and *DBR2* expression levels, with no significant change in *ALDH1* expression level (Fig. 5F–I). Notably, co-overexpression lines displayed even higher transcription levels of the key enzyme genes than either single-gene overexpression lines, supporting the notion that AaSnRK2.6 enhances the regulatory activity of AaORA in artemisinin biosynthesis.

Consistent with these transcriptional changes, HPLC analysis further demonstrated that OE-*AaSnRK2.6* alone led to a significant increase in artemisinin and DHAA accumulation relative to WT. Specifically, artemisinin content increased from 12.31 mg g^−1^ DW in WT to 14.44 mg g^−1^ DW in OE-*AaSnRK2.6* lines, while DHAA increased from 1.91 mg g^−1^ DW to 2.93 mg g^−1^ DW. Similarly, OE-*AaORA* lines accumulated 14.79 mg g^−1^ DW artemisinin and 3.07 mg g^−1^ DW DHAA. Importantly, the co-overexpression lines showed the highest metabolite accumulation, reaching 17.07 mg g^−1^ DW artemisinin and 4.28 mg g^−1^ DW DHAA, which were significantly higher than those in WT and both single-overexpression lines (Fig. 5J and K). These results indicate that AaSnRK2.6 alone positively regulates artemisinin biosynthesis, and that co-expression with *AaORA* produces a stronger enhancement effect.

### Knockdown of *AaSnRK2.6* does not significantly affect artemisinin biosynthesis

**Figure 6:**
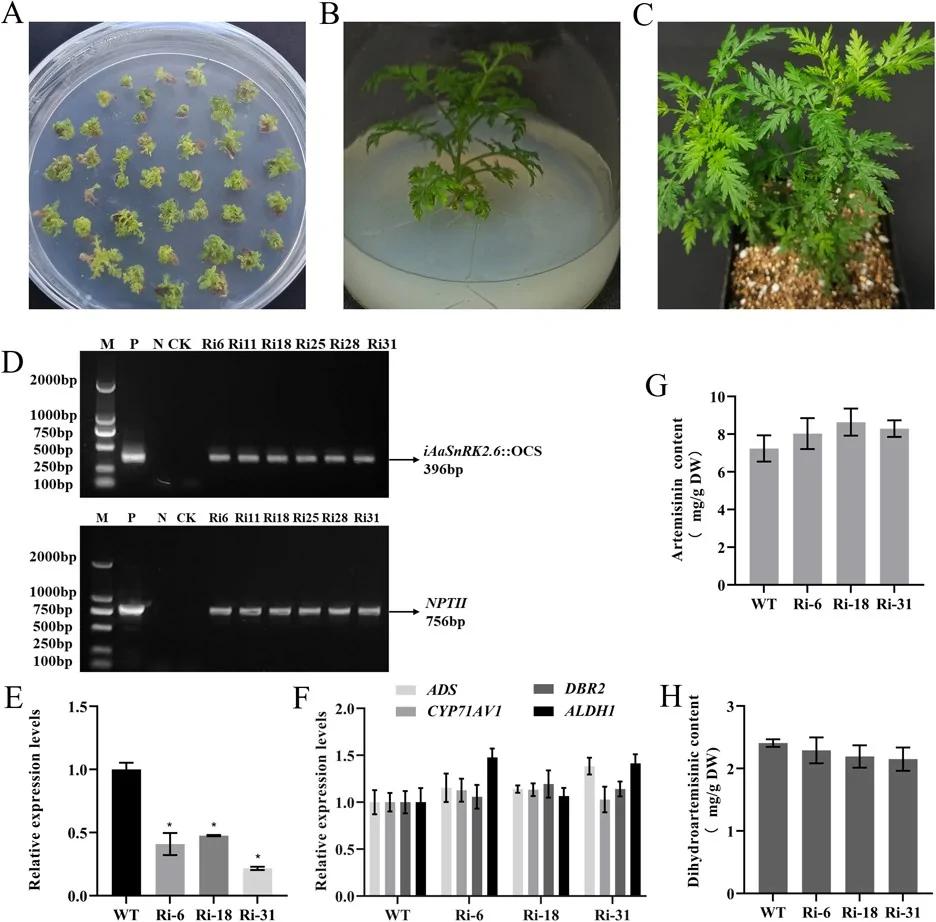
RNAi knockdown of *AaSnRK2.6* does not significantly affect artemisinin biosynthesis. (A–C) Regeneration of AaSnRK2.6-RNAi transgenic *A. annua* plants: screening for kanamycin resistance (A), root induction (B), and regenerated transgenic plants (C). (D) Genomic PCR analysis of putative transgenic lines. M: 2000 bp DNA marker; P: positive control; N: negative control; CK: blank control. (E) Relative expression levels of AaSnRK2.6 in transgenic lines as determined by qRT-PCR. (F) Expression levels of key artemisinin biosynthetic genes (*ADS*, *DBR2*, *ALDH1*) in RNAi lines. (G) Artemisinin content in RNAi lines. (H) Dihydroartemisinic acid content in RNAi lines. WT: wild-type *A. annua*; Ri-6, Ri-18, Ri-31: *AaSnRK2.6*-RNAi transgenic lines. Data are presented as mean ± SD (n = 9).

RNA interference (RNAi) transgenic *A. annua* lines targeting *AaSnRK2.6* were generated (Fig. 6A–C) to further validate the effect of AaSnRK2.6 on artemisinin biosynthesis. Genomic PCR confirmed successful integration of the RNAi construct and the kanamycin resistance gene (*NPTII*), with expected bands (~400 bp) detected in transgenic lines but absent in WT controls (Fig. 6D).

qPCR analysis indicated that *AaSnRK2.6* transcript levels were reduced by 52.5%–78.4% in lines Ri-6, Ri-18, and Ri-3 (Fig. 6E). However, the expression of *ADS*, *CYP71AV1*, *DBR2*, and *ALDH1* remained largely unchanged (Fig. 6F). Consistently, HPLC measurements showed that artemisinin and dihydroartemisinic acid contents in RNAi lines were not significantly different from that in WT controls (Fig. 6G and H). These findings suggest that knockdown of *AaSnRK2.6* does not markedly affect artemisinin accumulation. To further investigate whether the weak phenotype of the *AaSnRK2.6*-RNAi lines was associated with transcriptional compensation by other AaSnRK2 family members, we examined the expression levels of *AaAPK1* (Aannua12121S727101), *AaSnRK2.2* (Aannua02702S329990), *AaSnRK2.3* (Aannua17791S810490), *AaSnRK2.4* (Aannua05209S490740), and *AaSnRK2.5* (Aannua14947S778390) in the RNAi background. qRT-PCR analysis showed that none of these genes displayed significant expression changes in *AaSnRK2.6*-RNAi plants relative to WT (Supplementary Fig. S3), suggesting that the weak RNAi phenotype is unlikely to be explained by obvious transcriptional compensation at the mRNA level. We further analyzed the tissue-expression patterns and MeJA responsiveness of these AaSnRK2 genes and found that they exhibited distinct but partially overlapping expression profiles (Supplementary Figs S4–S5). Together, these results support the possibility of partial functional overlap among kinases.

## Discussion

The SnRK2 family constitutes a pivotal group of serine/threonine protein kinases in plants, broadly participating in ABA-dependent and ABA-independent signaling pathways to regulate stress responses, development, and metabolism [33]. In model plants such as *Arabidopsis thaliana* and *Oryza sativa*, SnRK2 members have been systematically classified and functionally characterized, demonstrating key roles in osmotic stress responses, stomatal regulation, and ABA-mediated processes [34, 35]. By contrast, in medicinal plants, studies on SnRK2s have mainly focused on ABA signaling. For example, in *A. annua*, the SnRK2 III member AaAPK1 is induced by ABA and promotes artemisinin biosynthesis via phosphorylation of the TF AabZIP1 [29]. Whether SnRK2s participate in JA-mediated secondary metabolism, however, remained unclear.

Here, we identified a novel SnRK2 III member, AaSnRK2.6, from *A. annua*, which is distinctly responsive to MeJA, representing the first demonstration of a SnRK2 III kinase participating in JA signaling in a medicinal plant. Subcellular localization experiments revealed dual distribution in the cytoplasm and nucleus, suggesting bifunctional roles in cytoplasmic signal transduction and nuclear transcriptional regulation. Mechanistically, AaSnRK2.6 specifically interacts with the JA-responsive TF AaORA in the nucleus, as confirmed by Y2H, BiFC, and LCI assays. Dual-luciferase assays further demonstrated that AaSnRK2.6 enhances AaORA-mediated transcriptional activation of *ADS*, *DBR2*, and *ALDH1* promoters, and co-overexpression of *AaSnRK2.6* and *AaORA* in *A. annua* significantly upregulates the expression of these genes and increases artemisinin and dihydroartemisinic acid accumulation. These results support a role for AaSnRK2.6 in the regulation of artemisinin biosynthesis and suggest that it may participate in JA-related signaling through functional association with AaORA. Consistent with this interpretation, MeJA treatment induced a stronger transcriptional and metabolic response in *AaSnRK2.6*-overexpression plants than in WT, providing additional physiological support for the involvement of AaSnRK2.6 in MeJA-responsive artemisinin biosynthesis.

Interestingly, the enhancement observed in the transient dual-luc assay was more modest than the stronger transcriptional changes detected in stable transgenic *A. annua* plants. This discrepancy likely reflects differences between the heterologous transient expression system in *N. benthamiana* and the native regulatory context of *A. annua*. In particular, the transient system may lack endogenous cofactors, species-specific regulatory components, or the full post-translational regulatory machinery required for stronger activation, whereas stable transgenic *A. annua* plants provide the native cellular background for a more complete regulatory output. The *in vitro* phosphorylation evidence further supports the idea that post-translational modulation may contribute to the stronger in planta effect. The dual localization of AaSnRK2.6 suggests that its cytoplasmic fraction may participate in relaying upstream JA signals, whereas nuclear AaSnRK2.6 potentially interacts with AaORA to modulate transcription. Such phosphorylation-dependent regulation of TFs is well documented in other systems, where phosphorylation can enhance DNA-binding affinity, promote nuclear translocation, or increase transcriptional activation capacity [36, 37]. Importantly, the *in vitro* kinase assay demonstrated that AaSnRK2.6 directly phosphorylate AaORA, providing biochemical support for the proposed kinase-TF regulatory relationship. However, the precise phosphorylation site(s), the requirement of AaSnRK2.6 kinase activity for this regulatory effect, and the *in vivo* functional consequences of AaORA phosphorylation remain to be determined.

Furthermore, RNAi-mediated knockdown of *AaSnRK2.6* did not significantly alter artemisinin content or target gene expression. qRT-PCR analysis showed that the other tested *AaSnRK2* family members were not significantly upregulated in the *AaSnRK2.6*-RNAi background, indicating that the weak phenotype of AaSnRK2.6-RNAi plants cannot be attributed to obvious transcriptional compensation within the AaSnRK2 family at the transcriptional level. Nevertheless, AaORA may also be regulated by kinases other than AaSnRK2.6. AP2/ERF TFs, including AaORA, are important regulators of plant secondary metabolism [38]. Some members of this family have also been reported to participate in kinase-mediated regulatory modules. For example, the AP2/ERF TF RAP2.6 is associated with both SnRK2.6 and CDK8 in *Arabidopsis* ABA signaling and drought responses [39]. In addition, an AP2/ERF TF named ORCA in *Catharanthus roseus* acts downstream of a MAPK cascade to modulate terpenoid indole alkaloid biosynthesis [26, 40]. Therefore, we cannot exclude the possibility that the weak phenotype of *AaSnRK2.6*-RNAi plants may involve broader kinase-mediated regulation at the protein or phosphorylation level, although direct evidence for this possibility remains to be obtained in future studies.

It is also important to consider the relationship between AaSnRK2.6 and the previously reported SnRK2 family member AaAPK1. Both kinases are responsive to ABA and MeJA and positively regulate artemisinin biosynthesis, suggesting that SnRK2-mediated control is an important feature of hormone-responsive metabolic regulation in *A. annua*. However, the two kinases appear to act through different transcription factor partners: AaAPK1 was reported to phosphorylate AabZIP1 [29], whereas our results show that AaSnRK2.6 interacts with and phosphorylates AaORA *in vitro*. These observations suggest a degree of functional diversification within the AaSnRK2 family, in which distinct SnRK2 members may converge on artemisinin biosynthesis through different downstream transcriptional modules. Whether AaAPK1 and AaSnRK2.6 share additional substrates or act in partially overlapping pathways remains to be determined.

Comparative analysis suggests that the role of AaSnRK2.6 in JA signaling exemplifies a broader mechanism by which specific SnRK2 kinases modulate secondary metabolism through targeted TF interactions. This expands our understanding of SnRK2 function beyond canonical ABA responses and underscores the potential for cross-talk between JA and ABA pathways in medicinal plants. Moreover, the synergistic action of AaSnRK2.6 and AaORA provides a promising target for metabolic engineering strategies aimed at improving artemisinin yield [41, 42]. Future studies should identify the relevant phosphorylation site(s) on AaORA, test the requirement of AaSnRK2.6 kinase activity using kinase-dead mutants, investigate additional interacting partners. High-throughput phosphoproteomic and transcriptomic approaches may further reveal the broader regulatory network in which AaSnRK2.6 operates.

Although our protein interaction, *in vitro* phosphorylation, transcriptional activation, and co-overexpression data strongly support a direct functional association between AaSnRK2.6 and AaORA, these results do not by themselves definitively establish the *in vivo* genetic hierarchy between the two regulators. Formal epistasis analysis using stable genetic combination lines, such as loss-of-function of *AaORA* in an *AaSnRK2.6*-overexpression background or overexpression of *AaORA* in *AaSnRK2.6* loss-of-function plants, will be required to rigorously determine whether AaORA is genetically required for the artemisinin-promoting effect of AaSnRK2.6. These experiments will be an important focus of future work.

**Figure 7:**
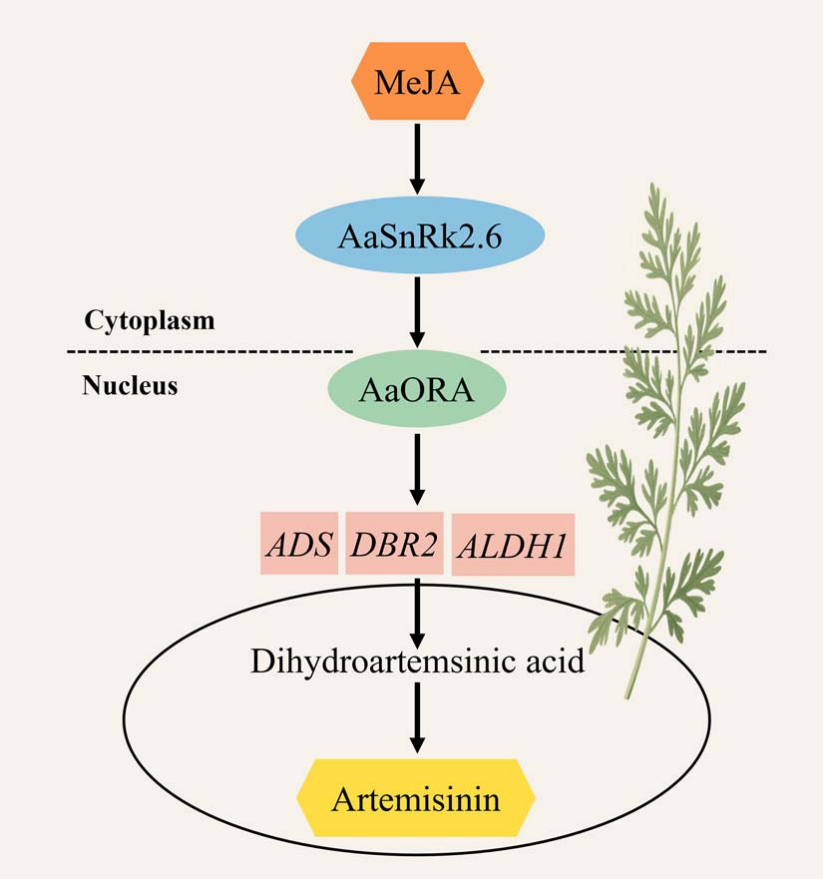
Proposed model of AaSnRK2.6-AaORA module in regulating artemisinin biosynthesis. MeJA and ABA induce AaSnRK2.6 expression. AaSnRK2.6 interacts with AaORA in the nucleus, enhancing activation of *ADS*, *DBR2*, and *ALDH1*, thereby promoting dihydroartemisinic acid and artemisinin accumulation.

In conclusion, this study provides the first evidence that a SnRK2 III kinase participates in JA signaling to regulate secondary metabolism in *A. annua* (Fig. 7). The synergistic action of AaSnRK2.6 and AaORA underscores a novel regulatory module that can be leveraged for rational improvement of artemisinin production. These findings not only enhance our mechanistic understanding of SnRK2-mediated JA signaling but also open new avenues for the bioengineering of medicinal plants with elevated artemisinin yields.

## Conclusions

In summary, we identified AaSnRK2.6 as a positive regulator of artemisinin biosynthesis in *A. annua*. *AaSnRK2.6* expression is induced by MeJA and ABA, and the protein localizes to both the cytoplasm and nucleus. AaSnRK2.6 physically interacts with AaORA and directly phosphorylates AaORA *in vitro*. Functional analyses showed that AaSnRK2.6 enhances AaORA-mediated activation of key biosynthetic genes, and that overexpression of *AaSnRK2.6* alone or together with *AaORA* significantly increased artemisinin and dihydroartemisinic acid contents, with the strongest effect observed in the co-overexpression lines. These findings support a kinase-transcription factor regulatory module involved in hormone-responsive artemisinin biosynthesis in *A. annua*.

## Materials and methods

### Plant material, growth conditions, ABA, and MeJA treatments

Wild-type (*Artemisia annua*) seeds were collected from plants cultivated at Southwest University (Chongqing, China). Seeds were surface-sterilized with 20% (v/v) sodium hypochlorite for 20 min, rinsed three times with sterile water, and germinated on half-strength Murashige and Skoog (MS) solid medium at 23 ± 2°C under a 16-h light/8-h dark photoperiod. Seedlings were transplanted into pots containing an organic substrate and maintained under the same environmental conditions.

*Nicotiana benthamiana* seeds were sown on solid medium and grown under identical environmental conditions. 35-day-old *N. benthamiana* plants were used for dual-luciferase, BiFC, and LCI assays.

For ABA and MeJA induction experiments, 45-day-old *A. annua* plants with uniform growth were sprayed with 10 μM ABA and 100 μM MeJA (dissolved in 0.5% ethanol). Leaves were collected at 0, 1, 3, 6, 9, and 12 h post-treatment, immediately frozen in liquid nitrogen, and pooled from three independent biological replicates for RNA extraction. For MeJA-response analysis in transgenic plants, WT, *AaSnRK2.6*-overexpression, and *AaSnRK2.6*-RNAi lines were treated with 100 μM MeJA. Leaf samples were collected at 6 h after treatment for gene-expression analysis, whereas samples for artemisinin determination were harvested after 8 d of treatment.

### Total RNA extraction and quantitative real-time PCR

Total RNA was extracted using the Total Plant RNA Kit (Tiangen, China) according to the manufacturer’s instructions and reverse-transcribed using the FastKing RT Kit (Tiangen, China). Quantitative real-time PCR (qRT-PCR) was performed with gene-specific primers (Table S1) under the following cycling conditions: 95°C for 3 min; followed by 40 cycles of 95°C for 10 s, 56°C for 20 s, and 72°C for 20 s. *β*-Actin was used as the internal reference gene [43]. Relative expression levels were calculated using the 2^−ΔΔCt^ method [44]. The same qRT-PCR procedure was used to analyze the expression of other AaSnRK2 family members in *AaSnRK2.6*-RNAi lines, in different tissues, and under MeJA treatment.

### Identification and phylogenetic analysis of *SnRK2* genes

SnRK2 amino acid sequences from *Arabidopsis thaliana* were retrieved from the TAIR database (www.arabidopsis.org) and used to construct hidden Markov model (HMM) profiles with HMMER v3.1 (http://hmmer.org/). Homologous sequences were identified by searching the *A. annua* protein database using an E-value threshold of 1 × 10^−9^. Redundant sequences were manually removed. Multiple sequence alignments were performed using ClustalW [45]. A maximum-likelihood phylogenetic tree was generated in MEGA version 5.0 with 1000 bootstrap replicates.

### Cloning of the candidate gene

First-strand cDNA was synthesized from 2 μg of total RNA isolated from young *A. annua* leaves. The coding sequence (CDS) of *AaSnRK2.6* was amplified using KOD DNA polymerase (Toyobo, Japan) under the conditions described previously [29]. The PCR products were ligated into the pJET2.1 vector (Thermo Fisher Scientific, USA) and sequenced for verification. All primers used in this study are listed in Supplementary Table S1.

### Subcellular localization of AaSnRK2.6

The CDS of *AaSnRK2.6* was inserted into the GFP-fusion vector pHB under the control of the CaMV 35S promoter using *PstI* and *SacI* restriction sites, generating the construct pHB-GFP-*AaSnRK2.6*. Recombinant plasmids were introduced into *Agrobacterium tumefaciens* strain GV3101-p19-psoup via the freeze–thaw method. Overnight bacterial cultures were resuspended in infiltration buffer (100 μM acetosyringone, 10 mM MES, pH 5.7, and MS liquid medium) to an OD_600_ of 0.6 and incubated in the dark at room temperature for 3 h. The suspensions were then infiltrated into fully expanded leaves of 35-day-old *N. benthamiana* plants. After 48 h, the leaves were stained with 1 mg/ml DAPI and observed using a confocal laser scanning microscope. GFP and DAPI signals were excited at 510 and 405 nm, respectively. GFP, DAPI, and bright-field images were collected and merged, with the scale bar uniformly set to 30 μm. All primers used in this study are listed in Supplementary Table S1.

### Y2H assay

The CDS of *AaSnRK2.6* was cloned into the pGBKT7 vector (bait), while the CDSs of jasmonate (JA)-responsive TF genes (*AaORA*, *AaERF1*, *AaERF2*, *AaGSW1*, *AaNAC1*, *AaWRKY1*, and *AaMYC2*) were individually cloned into pGADT7 (prey). Recombinant plasmids were co-transformed into *Saccharomyces cerevisiae* strain AH109 using the PEG3350/LiAc method. Briefly, yeast cells were grown in YPDA medium to an OD_600_ of 0.4, harvested, and sequentially washed in 1× TE buffer and 0.5× TE/1× LiAc solution. Competent cells were mixed with 1.5 μl each of bait and prey plasmids along with 10 μl of carrier DNA, followed by incubation with PEG3350 solution at 30°C for 30 min. After the addition of DMSO, the mixture was heat-shocked at 42°C for 7 min.

Cells were pelleted, resuspended in sterile water, and plated onto SD/-Trp/-Leu (-TL) selective medium. After incubation at 30°C for 2–3 days, single colonies were suspended in sterile water and spotted onto SD/-Trp/-Leu/-His (-TLH), SD/-Trp/-Leu/-His supplemented with 5 mM 3-AT, and SD/-Trp/-Leu/-His/-Ade (-TLHA) media. Growth was recorded after 2–3 days at 30°C to assess protein–protein interactions. All primers used in this study are listed in Supplementary Table S1.

### BiFC assay

The CDS of *AaSnRK2.6* was fused to the N-terminal fragment of YFP in the pSPYNE-35S vector, generating pSPYNE-35S-*AaSnRK2.6*, whereas AaORA was cloned into pSPYCE-35S to fuse with the C-terminal fragment of YFP, yielding pSPYCE-35S-*AaORA*. Constructs were individually introduced into GV3101 via the freeze–thaw method. Positive colonies were cultured overnight at 28°C in YEB medium supplemented with 50 mg/L kanamycin and 50 mg/L rifampicin at 200 rpm, adjusted to an OD_600_ of 0.6 in infiltration buffer (10 mM MES, pH 5.6, 20 μM acetosyringone), and incubated at room temperature for 3 h.

Equal volumes of *Agrobacterium* cultures carrying pSPYNE-35S-*AaSnRK2.6* and pSPYCE-35S-*AaORA* were mixed and co-infiltrated into fully expanded leaves of 35-day-old *N. benthamiana* plants. After 24 h in the dark, plants were transferred to a 16-h light/8-h dark photoperiod for 48–72 h. YFP fluorescence was observed using a confocal laser scanning microscope (excitation at 514 nm; emission at 527–552 nm). Three negative controls—pSPYNE-35S-*AaSnRK2.6* + pSPYCE-35S, pSPYCE-35S-*AaORA* + pSPYNE-35S, and pSPYNE-35S + pSPYCE-35S—were included to confirm the specificity of protein interaction and subcellular localization. All primers used in this study are listed in Supplementary Table S1.

### LCI assay

The CDS of *AaSnRK2.6* was fused to the N-terminal fragment of luciferase in pCAMBIA1300-cLUC, while *AaORA* was cloned into pCAMBIA1300-nLUC containing the C-terminal fragment. Recombinant constructs were introduced into *A. tumefaciens* GV3101. Positive colonies were cultured overnight at 28°C in YEB medium supplemented with 50 mg/L kanamycin and 50 mg/L rifampicin, adjusted to an OD_600_ of 0.6 in infiltration buffer (10 mM MES, pH 5.6, 10 mM MgCl₂, 150 μM acetosyringone), and incubated at room temperature for 3 h.

Equal volumes of *Agrobacterium* suspensions carrying nLUC and cLUC constructs were mixed and co-infiltrated into fully expanded leaves of 35-day-old *N. benthamiana* plants. After 12 h in the dark, plants were transferred to standard growth conditions (16-h light/8-h dark) for 48 h. Leaf tissues were infiltrated with 1 mM luciferin for 5 min, dark-adapted for 10 min, and luminescence was detected using a NightSHADE LB 985 imaging system with an exposure time of 1–5 min. Empty vector combinations served as negative controls. Interaction strength was quantified based on luminescence intensity. All primers used in this study are listed in Supplementary Table S1.

### Dual-luciferase assay

The CDS of *AaSnRK2.6* was cloned into the pHB-X-flag vector, which contains the CaMV 35S promoter and a Flag tag, to generate the effector construct. Promoter fragments of *ADS*, *CYP71AV1*, *DBR2*, and *ALDH1* were cloned upstream of the firefly luciferase gene in pGreen II 0800-LUC, serving as reporter constructs. Each effector and reporter combination, together with an empty pHB-GFP vector, was introduced into *A. tumefaciens* GV3101.

Positive colonies were cultured overnight at 28°C in LB medium containing the appropriate antibiotics, harvested by centrifugation, and resuspended in infiltration medium to an OD_600_ of 0.6 ± 0.05. *Acetosyringone* (100 mM, 1:500) and MES buffer (0.5 M, 1:50, pH 5.7) were added, and the mixtures were incubated for 3 h before infiltration into fully expanded leaves of *N. benthamiana*. For single-effector assays, *Agrobacterium* suspensions carrying the reporter, the corresponding effector, and the empty pHB-GFP vector were mixed in equal volumes (1:1:1). For co-expression assays, Agrobacterium suspensions carrying the reporter, AaSnRK2.6 effector, and AaORA effector were mixed in equal volumes (1:1:1). The empty pHB-GFP vector served as the negative control.

After 48 h under low-light conditions, leaf discs (2 cm in diameter) were collected and frozen in liquid nitrogen. Firefly (LUC) and Renilla (REN) luciferase activities were measured using the Dual-Luciferase® Reporter Assay System (Promega) according to the manufacturer’s instructions. Promoter activities are presented as relative LUC/REN ratios normalized to the empty-vector control, which represents the baseline promoter activity. All primers used in this study are listed in Supplementary Table S1.

### GST pull-down and *in vitro* kinase assays

To examine whether AaSnRK2.6 directly interacts with and phosphorylates AaORA *in vitro*, GST pull-down and *in vitro* kinase assays were performed using recombinant AaORA-GST, GST, and AaSnRK2.6-His proteins. For GST pull-down, GST served as the negative control. Equal amounts of recombinant proteins (50 μg per sample) were incubated with equilibrated GST Magarose Beads at 4°C for 4 h with gentle rotation. After washing three times with PBS, bound proteins were eluted and subjected to western blot analysis.

For the *in vitro* kinase assay, recombinant AaSnRK2.6-His and AaORA-GST were incubated in reaction mixtures containing ATP, 10× buffer, DTT, phosphatase inhibitor, and protease inhibitor at 30°C for 30 min. Reactions were terminated with 2× SDS-PAGE loading buffer and boiled at 95°C for 10 min before immunoblot analysis.

Pull-down samples were separated on 15% SDS-PAGE gels, whereas kinase assay samples were separated on 12% SDS-PAGE gels. After electrophoresis and transfer, membranes were blocked and incubated with the indicated primary antibodies overnight at 4°C. In the pull-down assay, anti-GST and anti-His antibodies were used to detect the bait and prey proteins, respectively. In the kinase assay, phosphorylation was detected using an anti-phosphoserine/threonine antibody, and AaORA-GST protein input was verified using an anti-GST antibody. Signals were visualized by ECL. Phos-tag detection was further used to support the phosphorylation of AaORA-GST by AaSnRK2.6-His.

### Generation of transgenic *A. annua* lines overexpressing *AaSnRK2.6*/*AaORA* or silencing *AaSnRK2.6*

For co-overexpression, the CDSs of *AaSnRK2.6* and *AaORA* were sequentially cloned into the pZZG100 binary vector to construct pZZG100-*AaSnRK2.6*-*AaORA*, which was introduced into *A. tumefaciens* strain EHA105. For RNA interference (RNAi), a specific fragment of *AaSnRK2.6* was inserted in both sense and antisense orientations into the pHANNIBAL vector and subsequently subcloned into pBIN19 to generate pBIN19-i*AaSnRK2.6*, also introduced into EHA105.

Leaf-disc transformation of sterile *A. annua* explants was performed following the method described previously [46]. After co-cultivation, explants were transferred to shoot induction medium supplemented with either 8 mg/L hygromycin (for overexpression) or 25 mg/L kanamycin (for RNAi) together with 200 mg/L cefotaxime. Rooting was induced on medium containing 0.05 mg/L NAA. Regenerated plants were screened by PCR using gene-specific primers targeting *AaSnRK2.6*::NOS, *AaORA*::OCS, the hygromycin resistance gene (Hyg), i*AaSnRK2.6*::OCS, and the kanamycin resistance gene (NPTII), as appropriate. All primers used in this study are listed in Supplemental Table S1.

More than 10 independent PCR-positive T0 transgenic lines were obtained for the co-overexpression construct. Three representative lines showing relatively high expression levels of both *AaSnRK2.6* and *AaORA* were selected for subsequent molecular and metabolite analyses. In addition, more than 10 independent PCR-positive T0 RNAi lines were obtained, and three representative lines showing relatively strong reduction of *AaSnRK2.6* transcript levels were selected for further analyses.

### Extraction and quantification of artemisinin and dihydroartemisinic acid in transgenic *A. annua*

Leaves from 3-month-old wild-type (WT) and transgenic *A. annua* plants were harvested, dried at 40°C to constant weight, and ground into a fine powder. Approximately, 0.1 g of powdered tissue was extracted with 25 ml preheated petroleum ether at 60°C by sonication at 40°C and 80 Hz for 60 min, followed by an additional 20 min under the same conditions. The combined extracts were concentrated to dryness using rotary evaporation at 50°C and 100 rpm. The residue was re-dissolved in 1 ml HPLC-grade methanol, centrifuged at 12 000 rpm for 10 min, and filtered through a 0.22-μm organic membrane prior to analysis.

Artemisinin content was quantified by HPLC-ELSD on a SunFire C18 column using a mobile phase of 60% acetonitrile and 40% ultrapure water at a flow rate of 1 ml min^−1^ and a column temperature of 40°C. ELSD conditions were set at a nitrogen pressure of 320–350 kPa and a drift tube temperature of 60°C. The injection volume was 20 μl. DHAA was measured by HPLC-PDA on the same column using a mobile phase of acetonitrile and 0.4% phosphoric acid solution (7:3, v/v) at a flow rate of 1 ml min^−1^ and a column temperature of 30°C, with detection at 205 nm. Each analysis was performed with three biological replicates, and metabolite contents were expressed as mg g^−1^ dry weight. Standard curves were generated using authentic standards. The calibration equation for artemisinin was *y* = 1.5646*x* + 15.667 (*R* [2] = 0.9992), and that for dihydroartemisinic acid was *y* = 1.259*x* + 16.115 (*R*^2^ = 0.9944).

## Supplementary Material

Web_Material_uhag287

## Data Availability

All data is available within the manuscript and its supporting materials.

## References

[1] Greenwood B, Mutabingwa T. Malaria in 2002. Nature. 2002;415:670–2 11832954 10.1038/415670a

[2] Olsson ME, Olofsson LM, Lindahl A-L. et al. Localization of enzymes of artemisinin biosynthesis to the apical cells of glandular secretory trichomes of *Artemisia annua* L. Phytochemistry. 2009;70:1123–8 19664791 10.1016/j.phytochem.2009.07.009

[3] White NJ . Qinghaosu (artemisinin): the price of success. Science. 2008;320:330–4 18420924 10.1126/science.1155165

[4] Tu Y . The discovery of artemisinin (qinghaosu) and gifts from Chinese medicine. Nat Med. 2011;17:1217–20 21989013 10.1038/nm.2471

[5] Delabays N, Simonnet X, Gaudin M. The genetics of artemisinin content in *Artemisia annua* L. and the breeding of high yielding cultivars. Curr Med Chem. 2001;8:1795–801 11772351 10.2174/0929867013371635

[6] Towler MJ, Weathers PJ. Evidence of artemisinin production from IPP stemming from both the mevalonate and the nonmevalonate pathways. Plant Cell Rep. 2007;26:2129–36 17710406 10.1007/s00299-007-0420-x

[7] Schramek N, Wang H, Römisch-Margl W. et al. Artemisinin biosynthesis in growing plants of *Artemisia annua*. A 13CO2 study. Phytochemistry. 2010;71:179–87 19932496 10.1016/j.phytochem.2009.10.015

[8] Paddon CJ, Westfall PJ, Pitera DJ. et al. High-level semi-synthetic production of the potent antimalarial artemisinin. Nature. 2013;496:528–32 23575629 10.1038/nature12051

[9] Mercke P, Bengtsson M, Bouwmeester HJ. et al. Molecular cloning, expression, and characterization of amorpha-4,11-diene synthase, a key enzyme of artemisinin biosynthesis in *Artemisia annua* L. Arch Biochem Biophys. 2000;381:173–80 11032404 10.1006/abbi.2000.1962

[10] Ro DK, Paradise EM, Ouellet M. et al. Production of the antimalarial drug precursor artemisinic acid in engineered yeast. Nature. 2006;440:940–3 16612385 10.1038/nature04640

[11] Zhang Y, Teoh KH, Reed DW. et al. The molecular cloning of artemisinic aldehyde Delta11(13) reductase and its role in glandular trichome-dependent biosynthesis of artemisinin in *Artemisia annua*. J Biol Chem. 2008;283:21501–8 18495659 10.1074/jbc.M803090200

[12] Göpfert JC, MacNevin G, Ro DK. et al. Identification, functional characterization and developmental regulation of sesquiterpene synthases from sunflower capitate glandular trichomes. BMC Plant Biol. 2009;9:86 19580670 10.1186/1471-2229-9-86 PMC2715020

[13] Rydén AM, Ruyter-Spira C, Quax W. et al. The molecular cloning of dihydroartemisinic aldehyde reductase and its implication in artemisinin biosynthesis in *Artemisia annua*. Planta Med. 2010;76:1778–83 20486073 10.1055/s-0030-1249930

[14] Shen Q, Zhang L, Liao Z. et al. The genome of *Artemisia annua* provides insight into the evolution of Asteraceae Family and Artemisinin biosynthesis. Mol Plant. 2018;11:776–88 29703587 10.1016/j.molp.2018.03.015

[15] Zheng H, Fu X, Shao J. et al. Transcriptional regulatory network of high-value active ingredients in medicinal plants. Trends Plant Sci. 2023;28:429–46 36621413 10.1016/j.tplants.2022.12.007

[16] Maes L, van Nieuwerburgh FCW, Zhang Y. et al. Dissection of the phytohormonal regulation of trichome formation and biosynthesis of the antimalarial compound artemisinin in *Artemisia annua* plants. New Phytol. 2011;189:176–89 20874804 10.1111/j.1469-8137.2010.03466.x

[17] Xia J, Ma YJ, Wang Y. et al. Deciphering transcriptome profiles of tetraploid *Artemisia annua* plants with high artemisinin content. Plant Physiol Biochem. 2018;130:112–26 29982168 10.1016/j.plaphy.2018.06.018

[18] Chen T, Li Y, Xie L. et al. AaWRKY17, a positive regulator of artemisinin biosynthesis, is involved in resistance to pseudomonas syringae in *Artemisia annua*. Hortic Res. 2021;8:217 34593786 10.1038/s41438-021-00652-6 PMC8484609

[19] Fu X, Peng B, Hassani D. et al. AaWRKY9 contributes to light- and jasmonate-mediated to regulate the biosynthesis of artemisinin in *Artemisia annua*. New Phytol. 2021;231:1858–74 33973259 10.1111/nph.17453

[20] Coello P, Hey SJ, Halford NG. The sucrose non-fermenting-1-related (SnRK) family of protein kinases: potential for manipulation to improve stress tolerance and increase yield. J Exp Bot. 2011;62:883–93 20974737 10.1093/jxb/erq331

[21] Ramon M, Dang TVT, Broeckx T. et al. Default activation and nuclear translocation of the plant cellular energy sensor SnRK1 regulate metabolic stress responses and development. Plant Cell. 2019;31:1614–32 31123051 10.1105/tpc.18.00500 PMC6635846

[22] Liu X, Li Z, Hou Y. et al. Protein Interactomic analysis of SAPKs and ABA-inducible bZIPs revealed key roles of SAPK10 in Rice flowering. Int J Mol Sci. 2019;20:1427 30901838 10.3390/ijms20061427 PMC6471077

[23] Wang Y, Hou Y, Qiu J. et al. Abscisic acid promotes jasmonic acid biosynthesis via a 'SAPK10-bZIP72-AOC' pathway to synergistically inhibit seed germination in rice (*Oryza sativa*). New Phytol. 2020;228:1336–53 32583457 10.1111/nph.16774 PMC7689938

[24] Hasan MM, Liu XD, Waseem M. et al. ABA activated SnRK2 kinases: an emerging role in plant growth and physiology. Plant Signal Behav. 2022;17:2071024 35506344 10.1080/15592324.2022.2071024 PMC9090293

[25] Yamada Y, Sato F. Tyrosine phosphorylation and protein degradation control the transcriptional activity of WRKY involved in benzylisoquinoline alkaloid biosynthesis. Sci Rep. 2016;6:31988 27552928 10.1038/srep31988 PMC4995487

[26] Paul P, Singh SK, Patra B. et al. A differentially regulated AP2/ERF transcription factor gene cluster acts downstream of a MAP kinase cascade to modulate terpenoid indole alkaloid biosynthesis in *Catharanthus roseus*. New Phytol. 2017;213:1107–23 27801944 10.1111/nph.14252

[27] Li S, Wang W, Gao J. et al. MYB75 phosphorylation by MPK4 is required for light-induced anthocyanin accumulation in *Arabidopsis*. Plant Cell. 2016;28:2866–83 27811015 10.1105/tpc.16.00130 PMC5155340

[28] Jia Y, Bai Z, Pei T. et al. The protein kinase SmSnRK2.6 positively regulates phenolic acid biosynthesis in *salvia miltiorrhiza* by interacting with SmAREB1. Frontiers in Plant Sci. 2017;8:1384 10.3389/fpls.2017.01384 PMC5552723 28848585

[29] Zhang F, Xiang L, Yu Q. et al. ARTEMISININ BIOSYNTHESIS PROMOTING KINASE 1 positively regulates artemisinin biosynthesis through phosphorylating AabZIP1. J Exp Bot. 2018;69:1109–23 29301032 10.1093/jxb/erx444 PMC6019033

[30] Ma YN, Xu DB, Li L. et al. Jasmonate promotes artemisinin biosynthesis by activating the TCP14-ORA complex in *Artemisia annua*. Sci Adv. 2018;4:eaas9357 30627665 10.1126/sciadv.aas9357 PMC6317983

[31] Lu X, Zhang L, Zhang F. et al. AaORA, a trichofme-specific AP2/ERF transcription factor of *Artemisia annua*, is a positive regulator in the artemisinin biosynthetic pathway and in disease resistance to Botrytis cinerea. New Phytol. 2013;198:1191–202 23448426 10.1111/nph.12207

[32] Ma YN, Xu DB, Yan X. et al. Jasmonate- and abscisic acid-activated AaGSW1-AaTCP15/AaORA transcriptional cascade promotes artemisinin biosynthesis in *Artemisia annua*. Plant Biotechnol J. 2021;19:1412–28 33539631 10.1111/pbi.13561 PMC8313134

[33] Chen J, Chen M, Li H. et al. An ABA-responsive transcription factor SmbZIP38 positively regulates salvianolic acids biosynthesis and stress-associated morphogenesis of *salvia miltiorrhiza*. Horticultural Plant Journal, in press. 2025

[34] Fujii H, Verslues PE, Zhu JK. Arabidopsis decuple mutant reveals the importance of SnRK2 kinases in osmotic stress responses *in vivo*. Proc Natl Acad Sci USA. 2011;108:1717–22 21220313 10.1073/pnas.1018367108 PMC3029766

[35] Son S, Park SR. The rice SnRK family: biological roles and cell signaling modules. Front Plant Sci. 2023;14:1285485 38023908 10.3389/fpls.2023.1285485 PMC10644236

[36] Naqvi S, Martin KJ, Arthur JSC. CREB phosphorylation at Ser133 regulates transcription via distinct mechanisms downstream of cAMP and MAPK signalling. Biochem J. 2014;458:469–79 24438093 10.1042/BJ20131115

[37] Ma GL, Han Y, Li T. et al. Phosphorylation modification reverses the transcriptional inhibitory activity of CsMYB4a in tea plants (*Camellia sinensis*). Horticultural Plant Journal. 2025;11:406–16

[38] Chen Q, Li N, Cui X. et al. AP2/ERF transcription factors regulate the biosynthesis of terpenoids, phenolics, and alkaloids in plants. Hortic Res. 2025;13:uhaf280 41647723 10.1093/hr/uhaf280 PMC12871079

[39] Zhu Y, Huang P, Guo P. et al. CDK8 is associated with RAP2.6 and SnRK2.6 and positively modulates abscisic acid signaling and drought response in *Arabidopsis*. New Phytol. 2020;228:1573–90 32619295 10.1111/nph.16787

[40] Liu S, Xing M, Yin X. MAPK regulates secondary metabolism and abiotic stress in horticultural and medicinal plants. Hortic Res. 2025;13:uhaf350 41821683 10.1093/hr/uhaf350 PMC12977962

[41] Li Y, Yang Y, Li L. et al. Advanced metabolic engineering strategies for increasing artemisinin yield in *Artemisia annua* L. Hortic Res. 2024;11:uhad292 38414837 10.1093/hr/uhad292 PMC10898619

[42] Qamar F, Mishra A, Ashrafi K. et al. Increased artemisinin production in *Artemisia annua* L. by co-overexpression of six key biosynthetic enzymes. Int J Biol Macromol. 2024;281:136291. 39368573 10.1016/j.ijbiomac.2024.136291

[43] Wang W, Wang Y, Zhang Q. et al. Global characterization of *Artemisia annua* glandular trichome transcriptome using 454 pyrosequencing. BMC Genomics. 2009;10:465 19818120 10.1186/1471-2164-10-465 PMC2763888

[44] Rao X, Huang X, Zhou Z. et al. An improvement of the 2ˆ(−delta delta CT) method for quantitative real-time polymerase chain reaction data analysis. Biostat Bioinforma Biomath. 2013;3:71–85 PMCID: PMC4280562 25558171 PMC4280562

[45] Chen C, Wu Y, Li J. et al. TBtools-II: a "one for all, all for one" bioinformatics platform for biological big-data mining. Mol Plant. 2023;16:1733–42 37740491 10.1016/j.molp.2023.09.010

[46] Shu G, Tang Y, Yuan M. et al. Molecular insights into AabZIP1-mediated regulation on artemisinin biosynthesis and drought tolerance in *Artemisia annua*. Acta Pharm Sin B. 2022;12:1500–13 35530156 10.1016/j.apsb.2021.09.026 PMC9069397

