# Supplementary material for: AaSnRK2.6 interacts with AaORA to positively regulate artemisinin biosynthesis in Artemisia annua
Source: Hortic Res. 2026 Jul 7;13(9):uhag287. doi: 10.1093/hr/uhag287 (PMC13557925; doi:10.1093/hr/uhag287)
Supplement: Supplementary file 1 [file web_material_uhag287.zip › Supplementary Figure.docx]

Supplementary Figure S1. Amplification of the *AaSnRK2.6* coding region and its sequence. (A) Electrophoresis of PCR fragment *AaSnRK2.6*. (B) The coding sequence of *AaSnRK2.6* and its deduced amino acid sequence. M: DNA Marker (100-2000). The coding sequence and the deduced amino acid sequence were shown in capital letters in bold font and the stop code was marked with.

Supplementary Figure S2. MeJA responsiveness of WT, *AaSnRK2.6*-overexpression, and *AaSnRK2.6*-RNAi plants. (A) Relative expression levels of *ADS*, *DBR2*, and *ALDH1* in WT, OE-*AaSnRK2.6*, and Ri-*AaSnRK2.6* plants after treatment with 100 μM MeJA for 6 h. (B) Artemisinin contents in WT, OE-*AaSnRK2.6*, and Ri-*AaSnRK2.6* plants after treatment with 100 μM MeJA for 8 days. Data are presented as mean ± SD (n = 3). Different letters indicate significant differences at *P* < 0.05.

Supplementary Figure S3. Expression levels of other *AaSnRK2* family members in *AaSnRK2.6*-RNAi lines. Relative expression levels of *AaAPK1*, *AaSnRK2.2*, *AaSnRK2.3*, *AaSnRK2.4*, and *AaSnRK2.5* in WT and three independent *AaSnRK2.6*-RNAi lines (Ri-6, Ri-18, and Ri-31) were determined by qRT-PCR. Data are presented as mean ± SD (n = 3).

Supplementary Figure S4. Tissue-expression patterns of additional *AaSnRK2* genes in WT *A. annua*. Relative expression levels of *AaSnRK2.2* (A), *AaSnRK2.3* (B), *AaSnRK2.4* (C), and *AaSnRK2.5* (D) in different tissues of WT *A. annua* were determined by qRT-PCR. S, stem; L, leaf; F, flower; GST, glandular secretory trichomes. Data are presented as mean ± SD (n = 3). Different letters indicate significant differences at *P* < 0.05.

Supplementary Figure S5. Expression responses of additional *AaSnRK2* genes to MeJA treatment. Relative expression levels of *AaAPK1*, *AaSnRK2.1*, *AaSnRK2.2*, *AaSnRK2.3*, *AaSnRK2.4*, and *AaSnRK2.5* were analyzed by qRT-PCR after 100 μM MeJA treatment for 0, 3, 6, and 12 h. Data are presented as mean ± SD (n = 3). Asterisks indicate significant differences compared with the 0 h control (**P* < 0.05, *P* < 0.01).
