# Supplementary material for: AaSnRK2.6 interacts with AaORA to positively regulate artemisinin biosynthesis in Artemisia annua
Source: Hortic Res. 2026 Jul 7;13(9):uhag287. doi: 10.1093/hr/uhag287 (PMC13557925; doi:10.1093/hr/uhag287)
Supplement: Supplementary file 1 [file web_material_uhag287.zip › Supplementary Table.docx]

Supplementary Table S1. Sequences of primers used in this study.

| Primer names | sequences | Application |
| --- | --- | --- |
| AaSnRK2.6-inGFP-F | agctgtacaagCTGCTGCAGATGGATCGATTACCGG | Subcellular localization |
| AaSnRK2.6-inGFP-R | CTAGAGGATCATTCGAGCTCCATAGCATAAACGATTTC |  |
| AaSnRK2.6-inBD-F | CATGGAGGCCGAATTCATGGATCGATTACCGGGG | Y2H |
| AaSnRK2.6-inBD-R | GCAGGTCGACGGATCCTTACATAGCATAAACGATTT |  |
| AaORA-AD-F | GGAGGCCAGTGAATTCATGTTTGCTACTTGCA | Y2H |
| AaORA-AD-R | CGAGCTCGATGGATCCTCAAAAAAAAAAAAAGTCATC |  |
| AaMYC2-AD-F | GCCCCGGGATGACGATGAATATATGGAA | Y2H |
| AaMYC2-AD-R | GCCCCGGGATGACGATGAATATATGGAA |  |
| AaERF1-AD-F | CGGAATTCATGATGCAAATGCCTTCGTTTGG | Y2H |
| AaERF1-AD-R | CGGGATCCTTAACCACTAACGGCTTCACTC |  |
| AaERF2-AD-F | CGGAATTCATGTCTTTCACTAATGAATCTTC | Y2H |
| AaERF2-AD-R | CGGGATCCTCAATTTCTTCCGGCAGCCC |  |
| AabHLH112-AD-F | GTGAATTCATGTTGTCTAGAGTTAATAGTG | Y2H |
| AabHLH112-AD-R | ATGGATCCTCAAGCGACACCATGGTAACC |  |
| AaGSW1-AD-F | GCGAATTCATGGATAACCAGCTAGATGC | Y2H |
| AaGSW1-AD-R | GCGTCGACCTAGCTCGAAGAATATATTTG |  |
| *AaNAC1-AD-F* | GAATTCATGAGTTCGTGTGAGAATTTGG | Y2H |
| *AaNAC1-AD-R* | GCGTCGACTCACATTTGAAAGGGC |  |
| AaWRKY1-AD-F | GCGAATTCATGGAAAGTGTTTGTGTTTATG | Y2H |
| AaWRKY1-AD-R | GCGGATCCTTAAAATTTGAAATCAAGGTCT |  |
| AaSnRK2.6-incLUC-F | TACGCGTCCCGGGGCGGTACCATGGATCGATTACCGG | LCI assay |
| AaSnRK2.6-incLUC-R | AAGCTCTGCAGGTCGACCATAGCATAAACGATTTCAC |  |
| AaORA-innLUC-F | AGAACACGGGGGACGAGCTCATGTTTGCTACTTGCATTCGCAC | LCI assay |
| AaORA-innLUC-F | ACGAGATCTGGTCGACAAAAAAAAAAAAGTCATCATC |  |
| AaORA-inflag-F | gcttgatatcgaattcATGTTTGCTACTTGCATTCG | Overexpression plasmited |
| AaORA-inflag-R | cgtcactagtggatccTCAAAAAAAAAAAAAGTCAT |  |
| RNAi-AaSnRK2.6-F | GCAAGCTTAAATCATGCAGATAATCGC | RNAi assay |
| RNAi-AaSnRK2.6-R | GCTCTAGAAGTTATACAATCATAATCCATCT |  |
| OCS-R | GCACAACAGAATTGAAAGCA | PCR assay |
| Nos-R | CGCGGTGTCATCTATGTTAC |  |
| Kan-F | ATGATTGAACAAGATGGATT | PCR assay |
| Kan-R | TCAGAAGAACTCGTCAAGAA |  |
| qActin-F | CCAGGCTGTTCAGTCTCTGTAT | qRT-PCR assay |
| qActin-R | CGCTCGGTAAGGATCTTCATCA |  |
| qADS-F | GGGAGATCAGTTTCTCATCTATGAA | qRT-PCR assay |
| qADS-R | CTTTTAGTAGTTGCCGCACTTCTT |  |
| qCYP71AV1-F | ACTGACCACTTCCATTGCTCTTG | qRT-PCR assay |
| qCYP71AV1-R | ACTTTCTGGCTAAATCCCTAACCC |  |
| qDBR2-F | ATCATCAACAAGCAAGCCCATTTC | qRT-PCR assay |
| qDBR2-R | GCGATAGTCTTCAACCACCTCTAG |  |
| qALDH1-F | CGGAGTAGTTGGTCACATCATTC | qRT-PCR assay |
| qALDH1-R | TTAATCACGCCATCAGGAACAC |  |
| QAaAPK1-F | AGGAGCCCGATCAACCTATG | qRT-PCR assay |
| QAaAPK1-R | CAGAGTCAAAATCGTCCAGATC |  |
| qAaSnRK2.2-F | CAATGGACCAGTTTGGGGGA | qRT-PCR assay |
| qAaSnRK2.2-R | GGTTGAGGTTGTTAGCCCCA |  |
| qAaSnRK2.3-F | AAACAGCTGCTCTCACGGAT | qRT-PCR assay |
| qAaSnRK2.3-R | ATGATGGCGGTGGGTTTCTT |  |
| qAaSnRK2.4-F | TCTTGGGTCGGGGAACTTTG | qRT-PCR assay |
| qAaSnRK2.4-R | CAAAGAGCTCTCCACCAGCA |  |
| qAaSnRK2.5-F | ACGCCTGCCTATATTGCTCC | qRT-PCR assay |
| qAaSnRK2.5-R | GGGTAAGCTCCCACAAGCAT |  |
| qAaSnRK2.6-F | AACCTCAACCAGTATCTC | qRT-PCR assay |
| qAaSnRK2.6-R | ATTACACCACTACTATCAATATC |  |
